# The Use and Utility of Adjuvant Checkpoint Inhibitors in Elderly Patients with Melanoma: A Single Institution Experience

**DOI:** 10.3390/cancers18121893

**Published:** 2026-06-10

**Authors:** Maira A. Bhatty, Natalie N. Chakraborty, Kevin G. Zablonski, Jarred M. Boone, Hailey Seibert, Trisha Lal, Hanna Kakish, Madelyn N. Stevens, Iris Y. Sheng, Andrew N. Hanna, Ankit Mangla, Richard S. Hoehn, Luke D. Rothermel

**Affiliations:** 1 School of Medicine, Case Western Reserve University, Cleveland, OH 44106, USA; natalie.chakraborty@uhhospitals.org (N.N.C.); kevin.zablonski@uhhospitals.org (K.G.Z.); hailey.seibert@uhhospitals.org (H.S.); trisha.lal@uhhospitals.org (T.L.); iris.sheng@uhhospitals.org (I.Y.S.); andrew.hanna@uhhospitals.org (A.N.H.); ankit.mangla@uhhospitals.org (A.M.); richard.hoehn@uhhospitals.org (R.S.H.); luke.rothermel@uhhospitals.org (L.D.R.); 2 Department of Surgery, Division of Surgical Oncology, University Hospitals Cleveland Medical Center, Cleveland, OH 44106, USA; 3 Department of Hematology and Oncology, School of Medicine, Case Western Reserve University, Cleveland, OH 44106, USA; 4 Department of Surgery, John Hopkins University, Baltimore, MD 21218, USA; hkakish1@jh.edu; 5 Department of Otolaryngology-Head and Neck Surgery, University Hospitals Cleveland Medical Center, Cleveland, OH 44106, USA; madelyn.stevens@uhhospitals.org; 6 University Hospitals Seidman Cancer Center, Cleveland, OH 44103, USA; 7 Case Comprehensive Cancer Center, Cleveland, OH 44106, USA

**Keywords:** adjuvant immunotherapy, immune checkpoint inhibitors, elderly patients, older patients, malignant cutaneous melanoma, stage III melanoma

## Abstract

**Background**: Elderly patients have low utilization of adjuvant ICIs, although they achieve similar RFS benefits as younger patients. It remains unclear why elderly patients use adjuvant ICIs less frequently and whether toxicity impacts treatment utilization. **Methods**: Adult patients with stage III melanoma treated from 2017 to 2023 at a single academic cancer center were retrospectively identified. Multivariable logistic regressions evaluated the association of age with receipt of adjuvant ICIs and toxicity. RFS was assessed using Kaplan–Meier and multivariable Cox proportional hazards regression. **Results**: Among 240 patients, those aged ≥ 75 years were less likely to receive adjuvant ICI than those aged 18–74 years (aOR: 0.30; 95% CI: 0.11–0.80). Among 53 (22.1%) patients who did not receive adjuvant ICI, 58% declined treatment, 15% were not offered adjuvant ICI by provider, 9% had a comorbid autoimmunity, and 6% had another comorbidity. A total of 12% of patients aged 75–80 years old declined treatment versus 33% of those aged 80–90 years old. Older age was not associated with toxicity, treatment interruption, or discontinuation from adjuvant ICI. Adjuvant ICI was associated with low recurrence (aHR: 0.76; 95% CI: 0.63–0.92), whereas age ≥ 75 years was associated with higher recurrence risk (aHR: 1.79; 95% CI: 1.04–3.10) and low overall survival (aHR: 3.07; 95% CI: 1.43–6.57) compared to patients aged 18–74 years. **Conclusions**: Older patients with stage III melanoma were less likely to receive adjuvant ICIs, despite similar toxicity, treatment interruption, and discontinuation rates across age groups. Because adjuvant therapy is associated with lower recurrence risk, efforts to better understand and address age-related differences in treatment use are warranted.

## Simple Summary

The utility of adjuvant immune checkpoint inhibitors (ICI) in elderly patients with stage III melanoma and occult lymph node metastasis has not been studied sufficiently. We utilized a comprehensive dataset at a single large academic center to assess recurrence-free survival (RFS) benefit from adjuvant ICI and whether toxicity from adjuvant ICI impacts utilization of adjuvant ICI in the elderly population. Our analysis showed that older patients are less likely to use adjuvant ICI than younger patients, and that difference is not explained by treatment-related toxicity. Patient perspectives regarding the risks and benefits of treatment appear to influence treatment refusal. Future evaluation of patient-reported outcomes and utility analysis can help define the subset of elderly patients for whom adjuvant ICI may be most beneficial.

## 1. Introduction

Adjuvant use of immune checkpoint inhibitors (ICI) has consistently shown recurrence-free survival (RFS) benefit in patients with high-risk resected stage III melanoma [1]. The EORTC1325/KEYNOTE-054 trial demonstrated 1-year RFS benefit with adjuvant pembrolizumab [2], and this benefit was sustained at 5 years (55.4% with pembrolizumab group vs. 38.3% in the placebo) [3]. Similar RFS benefits were demonstrated in the CheckMate238 trial with adjuvant nivolumab (50% with nivolumab vs. 39% with ipilimumab), and the EORTC18071 trial for adjuvant Ipilimumab (40.8% with ipilimumab vs. 30.3% in the placebo) [4,5,6]. However, due to strict inclusion criteria, patients older than 75 years old are often excluded from clinical trials [7,8]. Analysis of the European Melanoma Registry found inferior RFS with adjuvant pembrolizumab than observed in clinical trials, citing that patients in the registry were older and had a higher proportion of stage IIIC and IIID disease [9]. With elderly patients >70 years old accounting for over 45% of new melanoma diagnoses, a disparity in the data is seen when the trials above include only 17–26% of patients >64 years of age [10]. An underrepresentation of older patients in these clinical trials limits our knowledge of the risks and benefits of adjuvant ICI in elderly melanoma patients.

Prior publications have reported no significant difference in RFS between elderly and young patients treated with adjuvant ICI [11,12,13]. It is postulated that the magnitude of RFS benefit from adjuvant ICI may be greater in patients aged ≥ 65 years than in those aged < 65 years due to the poorer prognosis of melanoma in elderly patients [14,15]. Despite the apparent benefits of adjuvant ICI, elderly patients utilize adjuvant immunotherapy less frequently than younger patients [16,17,18,19,20,21]. The probability of starting adjuvant treatment is estimated to be 26% lower in patients aged > 65 years, with the most common reasons for foregoing adjuvant ICI being age, fear of adverse events, and impaired quality of life [22].

The impact of adjuvant ICI on immunotherapy-related adverse events (irAEs) in elderly patients is not clearly understood. Various studies have shown that the toxicity from adjuvant ICI is similar in older and younger patients and that adjuvant ICI is well tolerated by the elderly [19,23,24,25,26,27,28,29,30,31,32,33]. Other studies have found worse toxicity profiles in elderly patients, with a greater proportion of elderly patients experiencing irAEs of grade ≥ 3 [12,34], including a significantly higher proportion of skin, renal, or bowel toxicity [35]. The impact of immune toxicity may also be greater for older patients. Elderly patients experience a greater likelihood of irAE-related adverse sequelae, including hospital admission, because of risk factors more prevalent in the elderly, like polypharmacy, comorbid conditions, functional status, cognition, social support, nutrition, and frailty [30,36,37,38,39,40]. Moreover, it can be challenging to manage irAEs in elderly patients due to risks associated with corticosteroid use and reduced physiological reserve [41]. Thus, some studies have found that elderly patients discontinue adjuvant ICI treatment more frequently due to irAEs [13,42,43].

Our current understanding of the utility of adjuvant ICI in the elderly population is limited due to inconsistent evidence regarding their toxicity profiles. In this context, we profiled our single-center experience at a National Cancer Institute (NCI)-designated Comprehensive Cancer Center, examining age-related patterns in adjuvant ICI receipt, treatment-related toxicity, and RFS among older patients with stage III melanoma. Clarifying these age-related patterns with our treatment utilization and outcomes analysis may help inform discussions about adjuvant treatment for older patients.

## 2. Materials and Methods

### 2.1. Study Cohort and Institutional Review Board Approval

We utilized cancer registry data from University Hospitals Seidman Cancer Center to retrospectively identify adult patients treated for stage III melanoma between January 2017 and December 2023. Patients were included in the study if they received either adjuvant single-agent anti-PD-1 immunotherapy (nivolumab or pembrolizumab) or no adjuvant therapy. Patients were excluded if they were concurrently treated with systemic therapy for a different malignancy, if they received adjuvant therapy other than anti-PD-1 monotherapy, or if they started adjuvant ICI more than 12 weeks postoperatively. Electronic Medical Records (EMR) with incomplete information regarding adjuvant ICI duration and tolerance were also excluded. Institutional Review Board (IRB) approval was obtained for this study at University Hospitals Cleveland Medical Center (IRB protocol: STUDY20200347).

### 2.2. Variables of Interest

The primary independent variable of interest was age, dichotomized into two subgroups: 18–74 and ≥75 years. Stage III melanoma was further categorized into stages IIIA with lymph node metastasis ≤ 1 mm, IIIA with lymph node metastasis > 1 mm, IIIB, IIIC, and IIID. Additional variables of interest included sex, Charlson–Deyo Comorbidity Index (CCI), Eastern Cooperative Oncology Group (ECOG) status, tumor location, ulceration, and lymphovascular invasion. Race was not included as a covariate because all patients in the cohort were Non-Hispanic White.

The primary endpoint was receipt of adjuvant immunotherapy, which was categorized as no adjuvant ICI received or receipt of anti-PD-1 monotherapy. Reasons for not receiving adjuvant ICI were recorded for those patients who did not receive systemic therapy. Two independent reviewers examined and confirmed the refusal of therapy to be documented in the EMR. We also assessed recurrence-free survival, which was defined as the time from surgery to local, in-transit, nodal basin, or distant recurrence. Patients were censored at death or last follow-up. Recurrences were typically biopsy-proven, with some distant recurrences diagnosed by imaging. Additional secondary endpoints included toxicity experienced from adjuvant ICI use. Toxicity was defined using the Common Terminology Criteria for Adverse Events (CTCAE) [44], and was grouped into grade 1–2 toxicity (low-grade toxicity), grade 3–4 toxicity (high-grade toxicity), or toxicity of any grade. Datapoints were also collected on whether adjuvant ICI use was paused or stopped due to toxicity from treatment.

### 2.3. Statistical Analysis

Descriptive statistics and univariate analyses, including chi-square test, were utilized to characterize the patients based on age. Multivariable logistic regression was utilized to assess the association between age group and receipt of adjuvant anti-PD-1 therapy, adjusting for prespecified covariates mentioned above, including sex, ECOG status, CCI, and tumor characteristics. Adjusted odds ratios (ORs) and 95% confidence intervals were reported for multivariable logistic regressions [45].

RFS was estimated using Kaplan–Meier methods and compared with Log-Rank tests. Adjusted hazard ratios (aHRs) and 95% confidence intervals (CIs) for RFS were estimated using multivariable Cox proportional hazards regression, including age group, receipt of adjuvant therapy, and the same prespecified clinicopathologic covariates. All tests were two-sided, with alpha < 0.05 considered statistically significant. All analyses were performed using Stata 17 (StataCorp LLC, College Station, TX, USA).

## 3. Results

**Figure 1:**
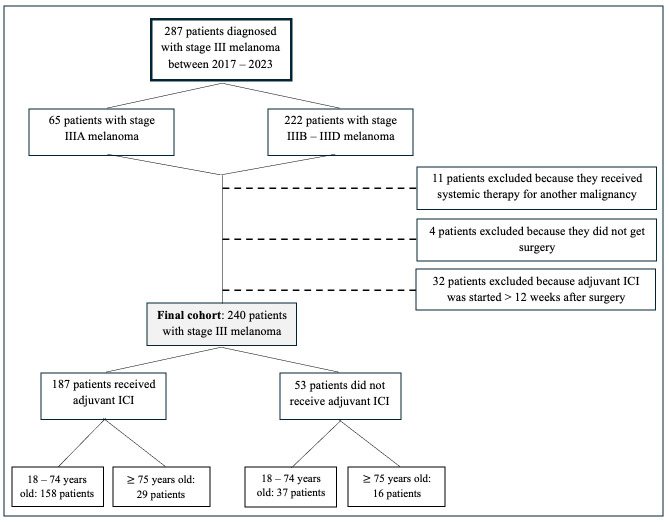
Flow Diagram for Patient Cohort.

240 patients were identified who were treated for stage III melanoma at our institution (Figure 1), of whom 195 (81.2%) were 18–74 years old and 45 (18.8%) were ≥75 years old. Eighty-one percent (*N* = 158) of patients aged 18–74 years old received anti-PD-1 monotherapy, compared with 64% (*N* = 29) of patients aged ≥ 75 years old (Table 1). A greater proportion of patients aged ≥ 75 years old had a CCI ≥ 3 (40%, versus 11.8% of patients aged 18–74 years old), while a greater proportion of patients aged 18–74 years had a CCI of 0 (54%, versus 26.7% of patients aged ≥ 75 years old) (*p* < 0.001). Seventy-seven percent of patients aged 18–74 years had an ECOG score of 0, compared to 49% of patients aged ≥ 75 years (*p* < 0.001). Trunk and extremities were the most common sites of melanoma for patients in both age groups. Most patients in both age groups had stage IIIC melanoma (Table 1).

### 3.1. Receipt of First-Line Adjuvant Immunotherapy

Older age over 75 was associated with lower anti-PD-1 therapy receipt compared to patients aged 18–74 years (aOR: 0.30; 95% CI: 0.11–0.80; Table 2). Additionally, stages IIIB (aOR: 7.19; 95% CI: 2.47–20.9), IIIC (aOR: 11.1; 95% CI:3.49–35.4) and IIID (aOR: 43.3; 95% CI: 2.85–659) were all associated with a higher likelihood of receiving first-line adjuvant ICI compared to stage IIIA with lymph node metastasis ≤ 1 mm. There were 53 patients (22.1%) in our cohort who were eligible and did not receive adjuvant ICI; among these, 58% of patients declined treatment (Table 3a). Other reasons for not being treated with first-line adjuvant ICI included ICI not being offered by the provider (15%), comorbid autoimmunity (9%), other comorbid condition (6%), and unknown reason (11%).

We also evaluated the utilization of first-line adjuvant ICI in elderly patients, in particular. Among 17 patients aged 75–80 years old, two were eligible but did not receive adjuvant ICI (Table 3b). Of these two patients, one patient did not receive adjuvant ICI because the patient declined treatment, and another patient was not recommended for treatment by the provider. Of 27 patients who were 80–90 years old, 9 patients were eligible but did not receive adjuvant ICI. Of these, 6 patients declined treatment with adjuvant ICI, one was not offered adjuvant ICI, and 2 had a comorbid condition that precluded them from utilizing adjuvant ICI. Overall, 12% patients (*N* = 2) aged 75–80 years old versus 33% patients (*N* = 9) aged 80–90 years old were eligible but did not receive adjuvant ICI. There was only one patient aged >90 years old in our cohort, and that patient received treatment with adjuvant ICI.

### 3.2. Recurrence-Free Survival (RFS)

**Figure 2:**
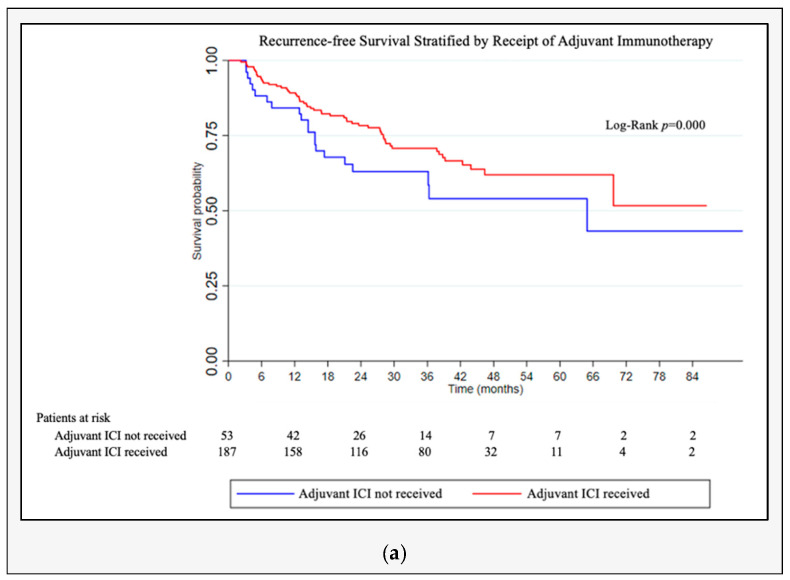

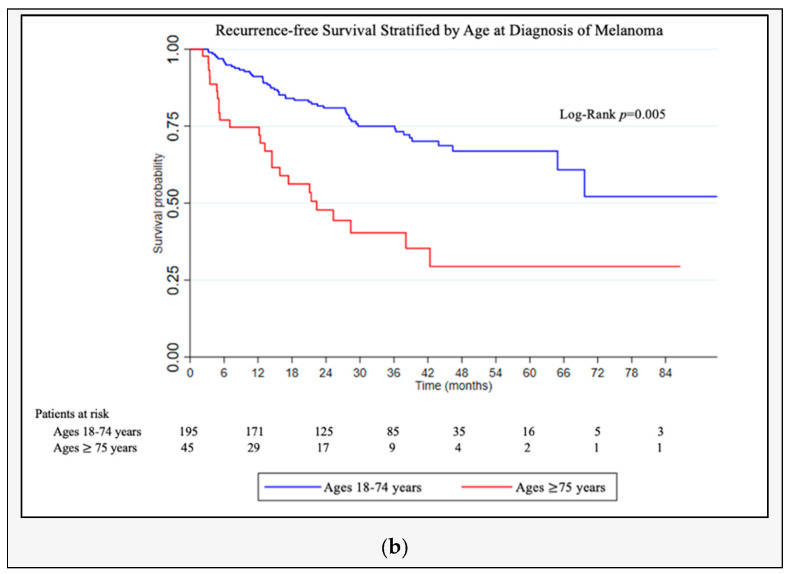
Kaplan–Meier Curves for Recurrence-Free Survival, Stratified by: (**a**) Receipt of Adjuvant Immunotherapy, and (**b**) Age of Diagnosis.

Kaplan–Meier curves demonstrated improved RFS among all patients who received first-line adjuvant ICI compared with no therapy (*p* = 0.005, Figure 2a), as well as improved RFS among patients aged 18–74 years versus those ≥75 years (*p* < 0.001, Figure 2b).

On multivariable Cox proportional hazards analysis, patients aged ≥ 75 years had a higher risk of melanoma recurrence than those aged 18–74 years (aHR: 1.79; 95% CI: 1.04–3.10; Table 4). Receipt of first-line adjuvant ICI led to a 24% lower risk of recurrence compared to no adjuvant therapy (aHR: 0.76; 95% CI: 0.63–0.92). Stage IIIA with lymph node metastasis > 1 mm (aHR 3.77, 95% CI: 1.05–13.6), stage IIIC (aHR 3.84, 95% CI: 1.47–10.0) and stage IIID (aHR 14.3, 95% CI: 3.72–54.6) were more likely to recur compared to stage IIIA with lymph node metastasis ≤1 mm. Lymphovascular invasion (aHR 2.74, 95% CI:1.50–5.00) was also associated with an increased likelihood of recurrence.

### 3.3. Overall Survival (OS)

On multivariable Cox regression results, patients aged ≥ 75 years had three times lower odds of overall survival compared to patients aged 18–74 years (aHR: 3.07; 95% CI: 1.43–6.57; Table 5). There was no significant benefit in overall survival with the receipt of adjuvant ICI (aHR: 0.91; 95% CI: 0.67–1.23). Head and neck tumors (aHR: 3.52; 95% CI: 1.61–7.73) and stage IIID (aHR: 29.0; 95% CI: 5.38–155) were associated with worse overall survival.

### 3.4. Toxicity from First-Line Adjuvant Immunotherapy

#### 3.4.1. Likelihood of Experiencing Toxicity

Forty-eight percent (*N* = 115) of the patients in our cohort experienced toxicity from adjuvant ICI. The three most common toxicities were hypothyroidism (25% (*N* = 20), of which 15% were aged ≥ 75 years), dermatitis (21% (*N* = 24), of which 17% were aged ≥ 75 years), and colitis (10% (*N* = 16), of which 19% were aged ≥ 75 years). Multivariable logistic regression showed no significant difference in the odds of experiencing any toxicity between patients aged 18–74 years and those aged ≥ 75 years (aHR: 1.16; 95% CI: 0.48–2.82; Table 6). Similarly, age was not a significant predictor of the odds of experiencing grade 1–2 or grade 3–4 toxicity from adjuvant ICI. Females were more likely than males to experience any toxicity (aOR 1.97; 95% CI: 1.07–3.63) or grade 3–4 toxicity (aOR 2.29; 95% CI: 1.08–4.85; Appendix A Table A1) from adjuvant ICI.

#### 3.4.2. Likelihood of Interruption in Treatment Due to Toxicity

Patients aged ≥ 75 years were as likely as patients aged 18–74 years old to pause treatment with adjuvant ICI due to any toxicity (aOR: 1.04; 95% CI: 0.48–2.25). There was also no significant difference between patients aged ≥ 75 years and patients aged 18–74 years old in the odds of pausing treatment with adjuvant ICI due to grade 1–2 or grade 3–4 toxicities. Patients with a CCI of 1 had over twice the odds of patients with a CCI of 0 to pause treatment with adjuvant ICI due to any toxicity (aOR: 2.37; 95% CI: 1.11–5.09) and grade 3–4 toxicity (aOR: 2.92; 95% CI: 1.15–7.38). Additionally, females were more than twice as likely as males to pause treatment due to grade 3–4 toxicity (aOR: 2.24, 95% CI: 1.01–5.00).

#### 3.4.3. Likelihood of Treatment Discontinuation Due to Toxicity

There were no significant differences between patients aged ≥ 75 years and those aged 18–74 years in the likelihood of stopping adjuvant ICI due to any toxicity or grade-specific toxicity. Females were three times as likely as males to discontinue treatment with adjuvant ICI due to grade 3–4 toxicity (aOR: 3.04; 95% CI: 1.11–8.29).

## 4. Discussion

In this retrospective single-center cohort of patients with stage III melanoma, older age was independently associated with lower receipt of adjuvant anti–PD-1 therapy. Among patients who did not receive adjuvant therapy, the most common documented reason was patient refusal, and this pattern appeared more frequent in the oldest subgroup. However, among treated patients, older age was not associated with higher odds of CTCAE toxicity, treatment interruption, or treatment discontinuation. In adjusted survival analyses, receipt of adjuvant therapy was associated with lower recurrence risk but no overall survival benefit, whereas age ≥ 75 years was associated with worse recurrence-free survival and overall survival. Together, these findings suggest that lower adjuvant ICI use in older patients may not be fully explained by observed treatment tolerance alone.

The lower use of adjuvant ICI in older patients is often attributable to patients’ perceptions of treatment risks and benefits. More than half of the patients in our cohort not treated with ICI, declined treatment because of potential side effects. Our results demonstrate that patients’ refusal of treatment with adjuvant ICI increases with age, with patients aged 80–90 years old more than 3 times as likely to decline treatment compared to patients aged 75–80 years old. Older patients did not experience higher risk of treatment CTCAE toxicity interruptions and were just as likely as young patients to experience toxicity from adjuvant ICI and to pause or stop treatment due to toxicities. However, prior studies have shown that toxicity in older patients may have a more significant impact due to risk factors like comorbidities, frailty, and social support [30,36,37,38]. Although we accounted for frailty and medical comorbidities in our analysis by including each patient’s CCI and ECOG in multivariable models, CCI and ECOG may not entirely capture frailty and social support required by elderly patients. This being a retrospective study was not able to interrogate the direct influence of these factors on patient choices. The concern about toxicities and the subsequent detrimental impact on quality of life may dissuade older patients from initiating treatment with adjuvant ICI, and this should be explored further in a qualitative manner [22].

The perception of benefits from adjuvant ICI may also differ among older patients. Although adjuvant ICI improves RFS in patients with melanoma, older patients might not obtain the same benefit from treatment as younger patients [9]. In fact, our analysis showed worse RFS for patients aged ≥ 75 years, which may be due to age-related decline in immune function, or immunosenescence. Immunosenescence can impact response to ICIs in the adjuvant setting, where limited tumor antigen exposure provides weaker T cell stimulation compared to the neoadjuvant or metastatic setting [46,47,48,49,50,51]. Moreover, the 9-year analysis of CheckMate238 trial and the 10-year analysis of COMBI-AD trial showed no overall survival benefit with adjuvant ICI [52,53], which may be complicated by crossover and subsequent-line therapies. The decision to pursue adjuvant ICI in elderly patients should be individualized based on life expectancy, functional status, patient values and recurrence consequences. Decreasing recurrence of melanoma may not be as important to older patients if life expectancy is limited, and RFS benefit may be weighed in the context of comorbid conditions or lower functional status. Conversely, if recurrence rates were decreased with therapy, there could be a benefit to minimizing the need for additional interventions, including biopsies, surgery, and systemic therapy upon recurrence. Future evaluation of patient-reported outcomes and performance of a utility analysis would help define the subset of elderly patients for whom adjuvant ICI may be most beneficial [14,15]. Moreover, image-recognizing Artificial Intelligence (AI) models have shown increasing promise in identifying high-risk visual and clinicopathologic patterns, including primary tumor location, stage burden, ulceration and other morphologic features that may correlate with recurrence risk [54,55,56]. Future multimodal AI systems may enable clinicians to integrate pathology, imaging, comorbidity, functional status and patient-reported preferences to better identify elder patients who would most benefit from immunotherapy.

Given that the elderly population is growing, it is imperative to better understand the utility of adjuvant ICI in older patients. The older population experiences increasing frailty and comorbidity burden, contributing to age being an exclusion criterion in many clinical trials [7,8]. The expansion of indications for adjuvant treatment to stage IIB and IIC creates a larger layer of complexity in choosing adjuvant ICI therapy [57,58]. Moreover, the question of performing sentinel node biopsy in the elderly to determine prognosis is becoming complicated and is being addressed in the ECOG EA6244 trial [59]. Nonetheless, it is essential to include representative samples of older patients in clinical trials in an effort to understand the benefits and risks of adjuvant ICI in this population. Until older cohorts are studied adequately, we will lack the data necessary to counsel older patients with melanoma regarding the use of adjuvant immunotherapy.

Our study also found gender-based differences in toxicity profiles of treatment with adjuvant ICI. Females in our cohort were more likely than males to experience any toxicity with adjuvant ICI, and to pause and discontinue treatment due to grade 3–4 toxicity. Females have previously been reported to experience a higher irAE burden, particularly endocrine disorders, resulting in more frequent treatment interruption than males [60,61,62,63]. This may be due to gender-specific differences in innate and adaptive immune responses, including a higher incidence of autoimmune conditions in females overall [60]. In comparison, some studies have reported no gender-based differences in irAEs with adjuvant ICI [62,64,65]. Nevertheless, similar to prior literature, this difference in irAEs did not translate into a difference in recurrence-free survival between males and females in our cohort.

There are several limitations to our study. First, the retrospective study design introduces potential selection bias and lead time bias. We countered this by carefully curating the data and having multiple observers verify its accuracy. Second, the study was not powered to evaluate the differential impact of adjuvant ICI in older versus younger patients; thus, it should be considered hypothesis-generating. Third, we lacked access to the financial implications of adjuvant therapy in the elderly population. A cost–benefit analysis in this population could assess potential financial toxicity associated with treatment, especially when third-party payment comes through Medicare/Advantage plans. Fourth, this study design did not allow for assessment of patient perspectives beyond what was reported in the medical record. Ultimately, a utility analysis that interviews patients to assess the benefits of potential outcomes at each decision point in the management of resected stage III melanoma would be the best way to qualitatively capture patients’ perspectives on the benefits of treatment versus observation. Finally, the lack of racial and ethnic diversity in our study limits generalizability to other populations where treatment preference patterns might be different. Future efforts should be aimed at multi-center studies to understand age-related treatment patterns in diverse populations.

## 5. Conclusions

In conclusion, while older patients are less likely to use adjuvant ICI than younger patients, that difference is not explained by differing CTCAE toxicity profiles experienced with treatment. Patient perspectives regarding the risks and benefits of treatment appear to influence treatment refusal. Real-world assessment of the survival benefits of adjuvant ICI and qualitative studies assessing patient perspectives on the risks and benefits of treatment are needed to fully understand whether low use of adjuvant ICI represents under-utilization in the elderly population.

## Figures and Tables

**Table 1:** Baseline Cohort Characteristics Stratified by Age of Diagnosis.

	Age 18–74 Years (*N* = 195)	Age 75+ Years (*N* = 45)	*p*-Value
	***n (%)***	***n (%)***	
Sex			0.490
*Male*	106 (54.4%)	27 (60.0%)	
*Female*	89 (45.6%)	18 (40.0%)	
Charlson Comorbidity Index			**<0.001**
*0*	106 (54.4%)	12 (26.7%)	
*1*	35 (17.0%)	9 (20.0%)	
*2*	6 (3.1%)	6 (13.3%)	
*3+*	23 (11.8%)	18 (40.0%)	
ECOG			**<0.001**
*0*	151 (77.4%)	22 (48.9%)	
*1*	35 (17.9%)	14 (31.1%)	
*2–4*	6 (3.1%)	5 (11.1%)	
*Unknown*	3 (1.5%)	4 (8.9%)	
Tumor Location			**0.002**
*Trunk or Extremities*	167 (85.6%)	30 (66.7%)	
*Head and Neck*	21 (10.8%)	14 (31.1%)	
*Other*	7 (3.6%)	1 (2.2%)	
Stage III			0.202
*III*	2 (1.0%)	1 (2.2%)	
*IIIA with lymph node metastasis ≤ 1 mm*	46 (23.6%)	4 (8.9%)	
*IIIA with lymph node metastasis > 1 mm*	10 (5.1%)	1 (2.2%)	
*IIIB*	52 (26.7%)	7 (15.6%)	
*IIIC*	81 (41.5%)	27 (60.0%)	
*IIID*	4 (2.1%)	5 (11.1%)	
Ulceration			0.960
*No*	104 (53.3%)	25 (55.6%)	
*Yes*	78 (40.0%)	17 (37.8%)	
*Unknown*	13 (6.7%)	3 (6.7%)	
Lymphovascular Invasion			0.690
*No*	161 (82.6%)	36 (80.0%)	
*Yes*	18 (9.2%)	6 (13.3%)	
*Unknown*	16 (8.2%)	3 (6.7%)	
Receipt of Adjuvant ICI			**0.016**
*No*	37 (19.0%)	16 (35.6%)	
*Yes*	158 (81.0%)	29 (64.4%)	

**Table 2:** Multivariable Logistic Regression For Odds of Receiving First-Line Adjuvant ICI.

Covariate	aOR (95% CI)	*p*-Value
Age at Diagnosis		
*18–74*	*Reference*	
*≥75*	**0.30 (0.11–0.80)**	**0.017**
Sex		
*Male*	*Reference*	
*Female*	1.14 (0.53–2.42)	0.743
Charlson Comorbidity Index		
*0*	*Reference*	
*1*	0.40 (0.15–1.06)	0.066
*2*	0.47 (0.16–1.38)	0.168
*≥3*	**0.30 (0.10–0.89)**	**0.030**
ECOG		
*0*	*Reference*	
*1*	0.61 (0.25–1.51)	0.288
*2–4*	0.25 (0.05–1.24)	0.091
*Unknown*	0.36 (0.06–2.13)	0.262
Tumor location		
*Trunk or Extremities*	*Reference*	
*Head and Neck*	1.10 (0.39–3.11)	0.862
*Other*	0.15 (0.01–2.88)	0.207
Stage III		
*IIIA with lymph node metastasis ≤ 1 mm*	*Reference*	
*IIIA with lymph node metastasis > 1 mm*	5.35 (0.85–33.6)	0.074
*IIIB*	**7.19 (2.47–20.9)**	**0.000**
*IIIC*	**11.1 (3.49–35.4)**	**0.000**
*IIID*	**43.3 (2.85–659)**	**0.007**
Ulceration		
*No*	*Reference*	
*Yes*	0.80 (0.30–2.13)	0.525
*Unknown*	**21.1 (1.24–359)**	**0.035**
Lymphovascular Invasion		
*No*	*Reference*	
*Yes*	1.28 (0.32–5.09)	0.967
*Unknown*	0.24 (0.04–1.37)	0.123

aOR = Adjusted Odds Ratio. CI = 95% Confidence Interval.

**Table 3:** (**a**) Reason for Not Receiving First-Line Adjuvant ICI. (**b**) Reason for Not Receiving First-Line Adjuvant ICI in Elderly Patients.

**(a)**						
	**Patient’s Choice**	**Not Offered by Physician**	**Comorbid Autoimmunity**	**Other Comorbid Condition**	**Unknown Reason**	
***N*** **(%)**	31 (58%)	8 (15%)	5 (9%)	3 (6%)	6 (11%)	**Total** = 53
**(b)**						
**Age Group**	**Total Number of Patients in Study**	**Patients Who Were Eligible but did not Receive Adjuvant ICI**				
		**Patient’s Choice**	**Not Offered by Physician**	**Other Comorbid Condition**		**Total**
		***N*** **(%)**	***N*** **(%)**	***N*** **(%)**		***N*** **(%)**
75–80 years	17	1 (6%)	1 (6%)	-		2 (12%)
80–90 years	27	6 (22%)	1 (4%)	2 (7%)		9 (33%)
>90 years	1	-	-	-		-

**Table 4:** Multivariable Cox Regression for Recurrence-Free Survival.

Covariate	aHR (95% CI)	*p*-Value
Age at Diagnosis		
*18–74*	*Reference*	
*≥75*	**1.79 (1.04–3.10)**	**0.037**
Sex		
*Male*	*Reference*	
*Female*	0.71 (0.43–1.16)	0.177
Tumor location		
*Trunk or Extremities*	*Reference*	
*Head and Neck*	1.58 (0.84–2.97)	0.158
*Other*	2.32 (0.45–11.9)	0.313
Stage III		
*IIIA with lymph node metastasis ≤ 1 mm*	*Reference*	
*IIIA with lymph node metastasis > 1 mm*	**3.77 (1.05–13.6)**	**0.043**
*IIIB*	0.98 (0.33–2.91)	0.967
*IIIC*	**3.84 (1.47–10.0)**	**0.006**
*IIID*	**14.3 (3.72–54.6)**	**0.000**
Ulceration		
*No*	*Reference*	
*Yes*	1.46 (0.83–2.59)	0.192
*Unknown*	0.55 (0.05–2.40)	0.273
Lymphovascular Invasion		
*No*	*Reference*	
*Yes*	**2.74 (1.50–5.00)**	**0.001**
*Unknown*	0.88 (0.24–3.30)	0.852
Receipt of Adjuvant ICI		
*No*	*Reference*	
*Yes*	**0.76 (0.63–0.92)**	**0.005**

aHR = Adjusted Hazards Ratio. CI = 95% Confidence Interval.

**Table 5:** Multivariable Cox Regression for Overall Survival.

Covariate	aHR (95% CI)	*p*-Value
Age at Diagnosis		
*18–74*	*Reference*	
*≥75*	**3.07 (1.43–6.57)**	**0.004**
Sex		
*Male*	*Reference*	
*Female*	1.06 (0.50–2.26)	0.876
Tumor location		
*Trunk or Extremities*	*Reference*	
*Head and Neck*	**3.52 (1.61–7.73)**	**0.002**
*Other*	2.85 (0.25–32.9)	0.402
Stage III		
*IIIA with lymph node metastasis ≤ 1 mm*	*Reference*	
*IIIA with lymph node metastasis > 1 mm*	4.31 (0.68–27.3)	0.121
*IIIB*	2.20 (0.52–9.31)	0.286
*IIIC*	2.76 (0.73–10.4)	0.136
*IIID*	**29.0 (5.38–155)**	**0.000**
Ulceration		
*No*	*Reference*	
*Yes*	0.65 (0.27–1.60)	0.352
*Unknown*	**7.44 (1.19–46.6)**	**0.032**
Lymphovascular Invasion		
*No*	*Reference*	
*Yes*	1.44 (0.43–4.78)	0.553
*Unknown*	**0.02 (0.001–0.47)**	**0.014**
Receipt of Adjuvant ICI		
*No*	*Reference*	
*Yes*	0.91 (0.67–1.23)	0.546

aHR = Adjusted Hazards Ratio. CI = 95% Confidence Interval.

**Table 6:** Multivariable Logistic Regression Analysis for Odds of Experiencing Toxicity, Odds of Pausing and Odds of Cessation of Adjuvant ICI Therapy due to Toxicity.

	Odds of Experiencing Any Toxicity			Odds of Pausing Treatment Due to Any Toxicity			Odds of Treatment Cessation Due to Any Toxicity		
Variable	*N*	aOR (95% CI)	*p*-Value	*N*	aOR (95% CI)	*p*-Value	*N*	aOR (95% CI)	*p*-Value
Age at Diagnosis									
*18–74*	33	*Reference*		30	*Reference*		18	*Reference*	
*≥75*	8	1.16 (0.48–2.82)	0.736	5	1.04 (0.48–2.25)	0.929	3	1.64 (0.71–3.77)	0.247
Sex									
*Male*		*Reference*			*Reference*			*Reference*	
*Female*		**1.97 (1.07–3.63)**	**0.029**		1.54 (0.87–2.74)	0.142		1.68 (0.89–3.17)	0.107
Charlson Comorbidity Index									
*0*		*Reference*			*Reference*			*Reference*	
*1*		1.55 (0.69–3.52)	0.291		**2.37 (1.11–5.09)**	**0.026**		1.97 (0.85–4.54)	0.114
*2*		1.15 (0.49–2.69)	0.751		1.41 (0.62–3.19)	0.412		1.40 (0.57–3.42)	0.458
*≥3*		0.83 (0.31–2.17)	0.700		2.33 (0.98–5.55)	0.056		2.45 (0.95–6.27)	0.063
ECOG									
*0*		*Reference*			*Reference*			*Reference*	
*1*		1.07 (0.49–2.31)	0.867		0.74 (0.36–1.53)	0.418		1.15 (0.53–2.49)	0.724
*2–4*		1.31 (0.28–6.09)	0.727		0.79 (0.21–3.00)	0.723		0.94 (0.22–3.93)	0.930
*Unknown*		-			0.56 (0.08–3.87)	0.556		0.88 (0.13–6.07)	0.893
Stage III									
*IIIA with lymph node metastasis ≤ 1 mm*		*Reference*			*Reference*			*Reference*	
*IIIA with lymph node metastasis > 1 mm*		1.24 (0.26–5.92)	0.788		**0.13 (0.02–0.70)**	**0.018**		**0.16 (0.03–0.88)**	**0.035**
*IIIB*		1.18 (0.45–3.13)	0.735		0.47 (0.21–1.07)	0.072		**0.27 (011–0.63)**	**0.003**
*IIIC*		0.94 (0.39–2.27)	0.889		**0.28 (0.13–0.59)**	**0.001**		**0.15 (0.07–0.34)**	**0.000**
*IIID*		1.06 (0.19–5.85)	0.950		0.25 (0.05–1.29)	0.097		**0.12 (0.02–0.78)**	**0.026**

aOR = Adjusted Odds Ratio. CI = 95% Confidence Interval. Dashes (-) denote variables with no observations in that subgroup.

## Data Availability

The datasets presented in this article are not readily available because of utilization of public health information and IRB oversight. Requests to access the dataset should be directed to Maira Bhatty, mab498@case.edu.

## Abbreviations

The following abbreviations are used in this manuscript:

CCI	Charlson–Deyo Comorbidity Index
ECOG	Eastern Cooperative Oncology Group
ICI	Immune Checkpoint Inhibitors
irAEs	Immunotherapy-related Adverse Events
RFS	Recurrence-free Survival

## Appendix A

**Table A1:** Multivariable Logistic Regression Analysis for Odds of Experiencing Toxicity, Odds of Pausing and Odds of Cessation of Adjuvant ICI Therapy due to Grade 3–4 Toxicity.

	Odds of Experiencing Grade 3–4 Toxicity		Odds of Pausing Treatment Due to Grade 3–4 Toxicity		Odds of Treatment Cessation Due to Grade 3–4 Toxicity	
Variable	aOR (95% CI)	*p*-Value	aOR (95% CI)	*p*-Value	aOR (95% CI)	*p*-Value
Age at Diagnosis						
*18–74*	*Reference*		*Reference*		*Reference*	
*≥75*	1.10 (0.41–2.96)	0.854	0.65 (0.20–2.09)	0.466	0.64 (0.16–2.66)	0.542
Sex						
*Male*	*Reference*		*Reference*		*Reference*	
*Female*	**2.29 (1.08–4.85)**	**0.030**	**2.24 (1.01–5.00)**	**0.048**	**3.04 (1.11–8.29)**	**0.030**
Charlson Comorbidity Index						
*0*	*Reference*		*Reference*		*Reference*	
*1*	**3.16 (1.30–7.67)**	**0.011**	**2.92 (1.15–7.38)**	**0.024**	1.57 (0.46–5.42)	0.474
*2*	1.81 (0.67–4.94)	0.245	1.42 (0.48–4.20)	0.529	1.42 (0.39–5.16)	0.597
*≥3*	0.74 (0.21–2.65)	0.646	0.77 (0.19–3.18)	0.720	1.42 (0.30–6.54)	0.659
ECOG						
*0*	*Reference*		*Reference*		*Reference*	
*1*	1.06 (0.43–2.60)	0.907	0.86 (0.32–2.31)	0.766	1.07 (0.33–3.42)	0.910
*2–4*	1.93 (0.41–9.18)	0.406	1.42 (0.25–8.17)	0.692	1.24 (0.13–12.2)	0.852
*Unknown*	-		-		-	
Stage III						
*IIIA with lymph node metastasis ≤ 1 mm*	*Reference*		*Reference*		*Reference*	
*IIIA with lymph node metastasis >1 mm*	0.84 (0.08–8.42)	0.882	0.99 (0.10–10.3)	0.996	1.30 (0.12–14.1)	0.827
*IIIB*	2.36 (0.76–7.37)	0.139	2.46 (0.73–8.29)	0.147	1.41 (0.34–5.90)	0.640
*IIIC*	2.13 (0.76–5.96)	0.152	2.00 (0.66–6.07)	0.224	1.28 (0.36–4.55)	0.708
*IIID*	2.00 (0.27–14.9)	0.497	3.42 (0.45–26.3)	0.236	1.95 (0.16–23.2)	0.598

aOR = Adjusted Odds Ratio. CI = 95% Confidence Interval. Dashes (-) denote variables with no observations in that subgroup.
